# Relationships Among Bone Morphological Parameters and Mechanical Properties of Cadaveric Human Vertebral Cancellous Bone

**DOI:** 10.1002/jbm4.10351

**Published:** 2020-03-12

**Authors:** Abeer Al‐Barghouthi, Seokgi Lee, Giovanni Francesco Solitro, Loren Latta, Francesco Travascio

**Affiliations:** ^1^ Department of Orthopaedic Surgery, Max Biedermann Institute for Biomechanics Mount Sinai Medical Center Miami Beach FL USA; ^2^ Department of Industrial Engineering University of Miami Coral Gables FL USA; ^3^ Department of Orthopaedic Surgery Louisiana State University Health Science Center‐Shreveport Shreveport LO USA; ^4^ Department of Orthopaedic Surgery University of Miami Miami FL USA

**Keywords:** MECHANICAL INDENTATION, μCT, HYDRAULIC PERMEABILITY, AGGREGATE MODULUS, BONE VOLUME FRACTION

## Abstract

Mechanical properties and morphological features of the vertebral cancellous bone are related to resistance to fracture and capability of withstanding surgical treatments. In particular, vertebral strength is related to its elastic properties, whereas the ease of fluid motion, related to the success of incorporation orthopedic materials (eg, bone cement), is regulated by the hydraulic permeability (K). It has been shown that both elastic modulus and permeability of a material are affected by its morphology. The objective of this study was to establish relations between local values of K and the aggregate modulus (H), and parameters descriptive of the bone morphology. We hypothesized that multivariate statistical models, by including the contribution of several morphology parameters at once, would provide a strong correlation with K and H of the vertebral cancellous bone. Hence, μCT scans of human lumbar vertebra were used to determine a set of bone morphology descriptors. Subsequently, indentation tests on the bone samples were conducted to determine local values of K and H. Finally, a multivariate approach supported by principal component analysis was adopted to develop predictive statistical models of bone permeability and aggregate modulus as a function of bone morphology descriptors. It was found that linear combinations of bone volume fraction, trabecular thickness, trabecular spacing, structure model index, connectivity density, and degree of anisotropy provide a strong correlation (*R* ^2^ ~ 76%) with K and a weaker correlation (*R* ^2^ ~ 47%) with H. The results of this study can be exploited in computational mechanics frameworks for investigating the potential mechanical behavior of human vertebra and to develop strategies to treat or prevent pathological conditions such as osteoporosis, age‐related bone loss, and vertebral compression fractures. © 2020 The Authors. *JBMR Plus* published by Wiley Periodicals, Inc. on behalf of American Society for Bone and Mineral Research.

## Graphical abstract

Bone morphological parameters predict mechanical properties of human vertebral cancellous bone.

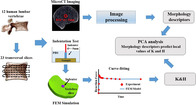

## Introduction

Knowledge of the structure–function relationships of the vertebral cancellous bone is crucial to understand its physiology and to develop strategies to treat or prevent pathological conditions such as osteoporosis, age‐related bone loss, and vertebral compression fractures.^1^, ^2^, ^3^ The cancellous bone of the vertebra is a porous solid structure saturated with marrow. Hence, its macroscopic mechanical behavior is usually described through the theory of poroelasticity.^4^

Mechanical properties and morphological features of the vertebral cancellous bone play a fundamental role in terms of resistance to fracture and the capability of withstanding a specific surgical treatment (eg, internal fixation versus bone cement augmentation).^5^ On one hand, bone apparent density has been associated with elastic modulus^6^, ^7^ and strength^8^; on the other, bone morphology has implications on the ease of fluid motion within the trabecula.^9^ The physical parameter quantifying the ease of fluid motion is the hydraulic permeability (K): It has been shown that it can determine the success of incorporation and vascularization of a bone graft or the integration of other orthopedic materials.^10^, ^11^

Using experimental or computational approaches focusing on entire anatomical structures such as the vertebral body, several studies correlated parameters describing bone density to modulus of elasticity^12^ and permeability.^9^, ^13^, ^14^ Even though, in material science, it has been shown that permeability is affected by morphology,^15^, ^16^ only Teo and Teoh attempted to find for trabecular bone a correlation between bone morphological descriptors and permeability.^17^ They measured trabecular bone permeability using a microscopic computational fluid dynamics approach and found that K could be predicted by the bone surface density with a correlation coefficient of 22%. Their study gave a first insight on the relevance of bone morphological parameters as predictors for hydraulic permeability. However, their experimental analysis was limited by the variability of the bone volume fraction of their specimens being equal to 0.149 ± 0.016. This parameter is considered to play a major role in influencing bone mechanical properties,^18^, ^19^ and exhibits significantly larger variations than the values explored in ref. 17^17^: For human trabecular bone, the bone volume fraction ranges from 0.076 to 0.258.^18^

In light of the limitations of previous studies and of the relevance that tissue structure has in determining bone properties, we used a poroelastic approach^20^ to establish the relations that exist between parameters descriptive of bone morphology and local values of aggregate elastic modulus (H) and K. With the purpose of realistically representing the pathophysiological variance of human bone morphology, we selected vertebral specimens covering a broad spectrum of morphological parameters. We hypothesized that multivariate statistical models, by including the contribution of several morphology parameters at once, would reveal a strong correlation with the mechanical properties of the vertebral cancellous bone.

## Materials and Methods

### Specimen description and preparation

Specimens were harvested from subjects deceased for pathologies not related to any musculoskeletal disease. Twelve whole human vertebras (L1 to L4) were obtained from fresh frozen human cadaver spines (age 57.8 ± 7.8 years; range 48 to 69 years; 40% female). Donors' cause of death was not related to musculoskeletal diseases. Samples' BMDs were 0.758 ± 0.348 g/cm^−2^, ranging from 0.215 to 1.187 g/cm^−2^. Such a broad range of BMD was purposefully included to test the sensitivity of the presented approach on a wide range of bone structural and compositional presentation. Following superior endplate removal, 5‐mm‐thickness slices were obtained with sequential cuts in the craniocaudal direction. To avoid the endplates, any slice containing portions of the endplates was excluded from the experiments. A maximum of three axial slices were obtained per vertebra and used for the experiments, resulting in a total of 23 samples (*n* = 23).

### μCT image acquisition and image analysis

A μCT scanner (SkyScan1176; Bruker BioSpin Corp., Manning Park, MA, USA) imaged the slices at 50 kV and 500 μA with 18‐μm resolution along the rostral‐caudal axis. The acquired images were analyzed via image analysis software ImageJ v1.52i in combination with the plugin BoneJ v1.4.3 to yield descriptors of the trabecular bone morphology.^21^ Specifically, the stacks of images obtained via μCT were binarized using the ImageJ default thresholding algorithm applied to each image of the stack. For each specimen, a circular region of interest (ROI) in 5‐mm diameter was extracted and six morphological parameters were measured: (i) the bone volume fraction (BV/TV), calculated as the number of foreground (bone) voxels divided by the total number of voxels in the image; (ii) mean trabecular thickness (Tb.Th); (iii) mean trabecular spacing (Tb.Sp), calculated using previously described image analysis methods^22^, ^23^; (iv) structure model index (SMI), according to the algorithm proposed by Hildebrand and Rüegsegger^23^; (v) connectivity density (Conn.D), and (vi) degree of anisotropy (DA), computed as previously reported.^24^, ^25^

### Indentation tests

Our μCT data indicated that space between trabecula ranged from 200 to 1500 mm (see the Results section). Following previously adopted proportions between pore sizes and indenters’ diameters,^26^ the vertebral slices were compressed by a single cylindrical indenter of 5‐mm diameter in correspondence with the ROI. Centering of the ROI was ensured by placing on the compression place, behind the bone slice, a printed 1:1 reproduction of the axial CT slice with the ROI marked. The indenter was connected to a servo‐electric testing system (Instron E3000; Instron, Norwood, MA, USA) with a 500‐N load cell. A stress‐relaxation test was performed by compressing the vertebral slice at 0.25 mm/s rate until reaching 5% compression (with respect to the unloaded height of the sample). The final displacement was held until reaching equilibrium (approximately 800 s), and the time‐dependent reaction force of the sample was measured at a sampling rate of 10 Hz. During testing, the samples were embedded in a phosphate buffer solution.

### Parameters estimation using finite element analysis

**Figure 1:**
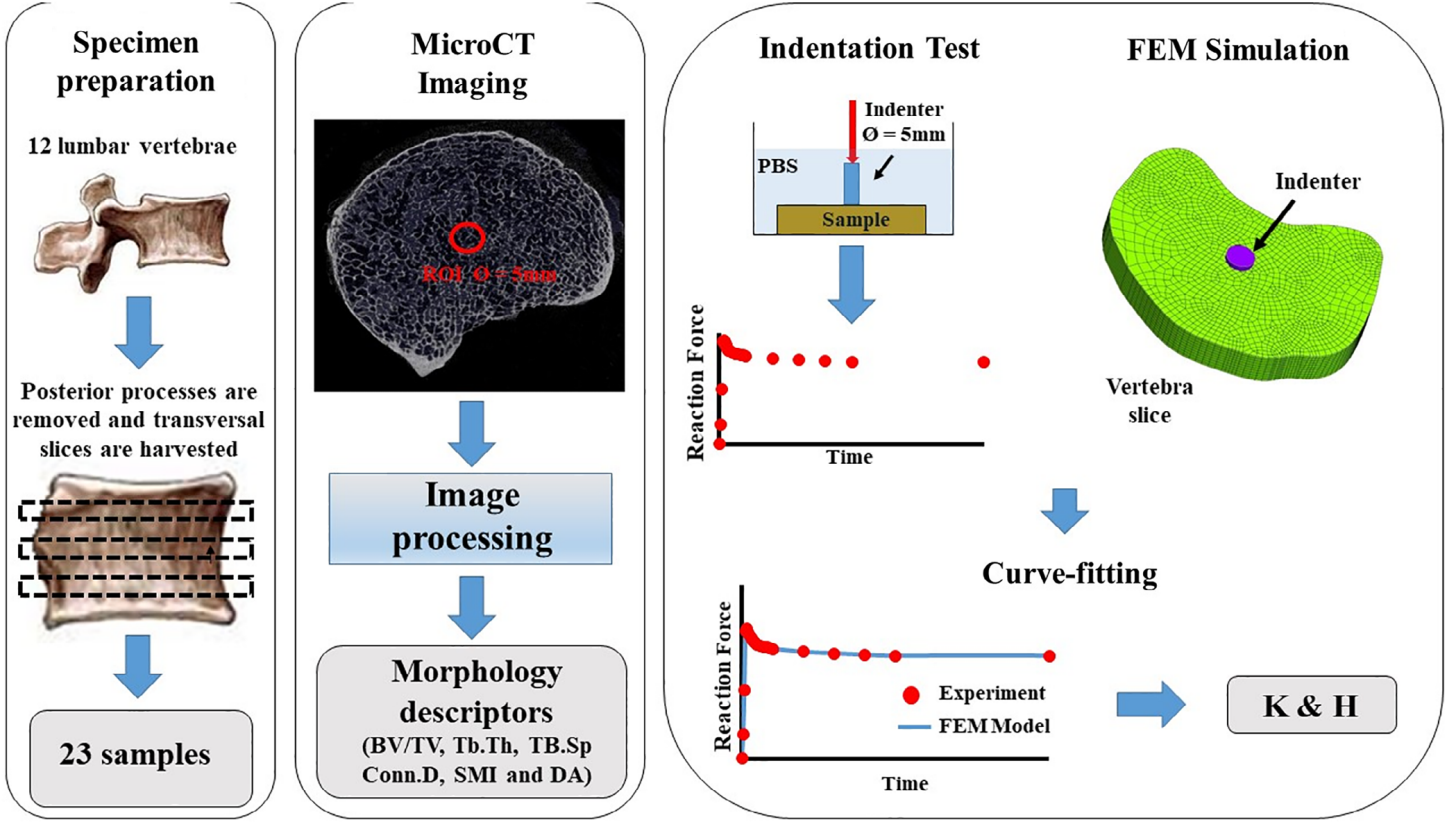
Schematic of the workflow for this study. Twenty‐three transversal slices of vertebral bodies were obtained from 12 lumbar vertebras. μCT scanning provided bone morphology descriptors. Curve‐fitting of indentation tests with results from Finite Element Method (FEM) simulations yielded values of K and E. BV/TV = bone volume over total volume; Conn.D = connectivity density; DA = degree of anisotropy; SMI = structure model index; Tb.Sp = trabecular spacing; Tb.Th = trabecular thickness.

Permeability and stiffness of the slices were quantified through inverse parameter estimation using finite element modeling of the experiments performed. The relaxations of the reaction force over time were curve‐fitted with the solution of finite element models simulating the indentation tests to yield K and H of the vertebral slices. The curve‐fitting was performed in the finite element modeling suite FEBio v2.6.2 (University of Utah, Salt Lake City, UT, USA)^27^ using the nonlinear least‐squares minimization of the objective function provided by the built‐in Levenberg–Marquardt algorithm. The bones were modeled as a biphasic continuum according to established theoretical frameworks.^20^, ^28^ Specifically, the material was characterized as an isotropic elastic noncompressible solid phase of modulus H embedded into an inviscid, incompressible fluid phase, where H is the aggregate elastic modulus according to the definition provided by Mow and colleagues.^20^ Fluid percolation through the vertebral slice was assumed to be governed by Darcy's law with constant isotropic permeability K. The geometry of the computational domain reflected the geometrical characteristics (shape and size) of the regions of interest previously tested. Approximately 20,000 hexahedral elements were used to discretize each vertebral slice and indenter. Mimicking experimental conditions, the bottom of the vertebral slice was assumed to be fixed and in contact with an impermeable surface (no fluid flux). The fluid gauge pressure on the remaining surface of the sample was set to zero. The indenter was modeled as a noncompressible solid with elastic properties similar to those of stainless steel. Vertical displacement of the indenter was prescribed to simulate the indentation at the same rate used for the experiments. A frictionless contact was imposed between the indenter and the vertebral slice.^29^ The output of the computer simulation was the time‐dependent reaction force on the indenter. The parameter optimization analysis to estimate the vertebral parameters K and H was carried out via a FEBio optimization module. A schematic of the experimental and computational workflow for this study is given in Fig. 1.

### Statistical analysis

All the experimental parameters determined in this analysis were reported in terms of mean ± SD. Preliminary univariate regression analyses were conducted to determine relationships between the dependent variables K and H, and the hypothesized predictors BV/TV, Tb.Th, Tb.Sp, SMI, Conn.D, and DA, if any. Additional univariate analyses were carried out to investigate if and how either the regressors (BV/TV, Tb.Th, Tb.Sp, SMI, Conn.D, and DA) or the dependent variable (H and K) were related to one another. Finally, aimed at investigating the predictive capabilities of all the regressors combined, a principal component analysis (PCA) was conducted to identify the variance–covariance structure of the given set of parameters and finally individuate linear combinations of six regressors. After investigating individuated properties of parameters by PCA, a multivariate regression analysis was finally conducted to show statistical relationships of vertebral bone features with respect to morphological characteristics of the vertebral samples. For all the analyses carried out, outliers were detected using the Mahalanobis squared distance.^30^

## Results

A summary of the descriptive statistics for the mechanical properties K and H, and the morphology descriptors of the vertebral samples is provided in Table 1.

**Table 1:** Descriptive Statistics for the Measured Experimental Parameters

Parameter	*n*	Mean	SD	Min	Max
K (m^2^)	23	3.21 × 10^−17^	5.13 × 10^−17^	1 × 10^−19^	2.2 × 10^−16^
H (MPa)	23	5.269	3.394	1.07	13.5
BV/TV	23	0.15	0.05	0.03	0.24
Tb.Th (μm)	23	151.2	38.51	102.34	254.9
Tb.Sp (μm)	23	545	298	202.3	1546.8
Conn.D (mm^−3^)	23	39.15	42.05	2.87	155.14
SMI	23	3.06	0.27	2.7	3.72
DA	23	1.66	0.22	1.24	2.21

BV/TV = bone volume over total volume; Conn.D = connectivity density; DA = degree of anisotropy; SMI = structure model index; Tb.Sp = trabecular spacing; Tb.Th = trabecular thickness.

**Figure 2:**
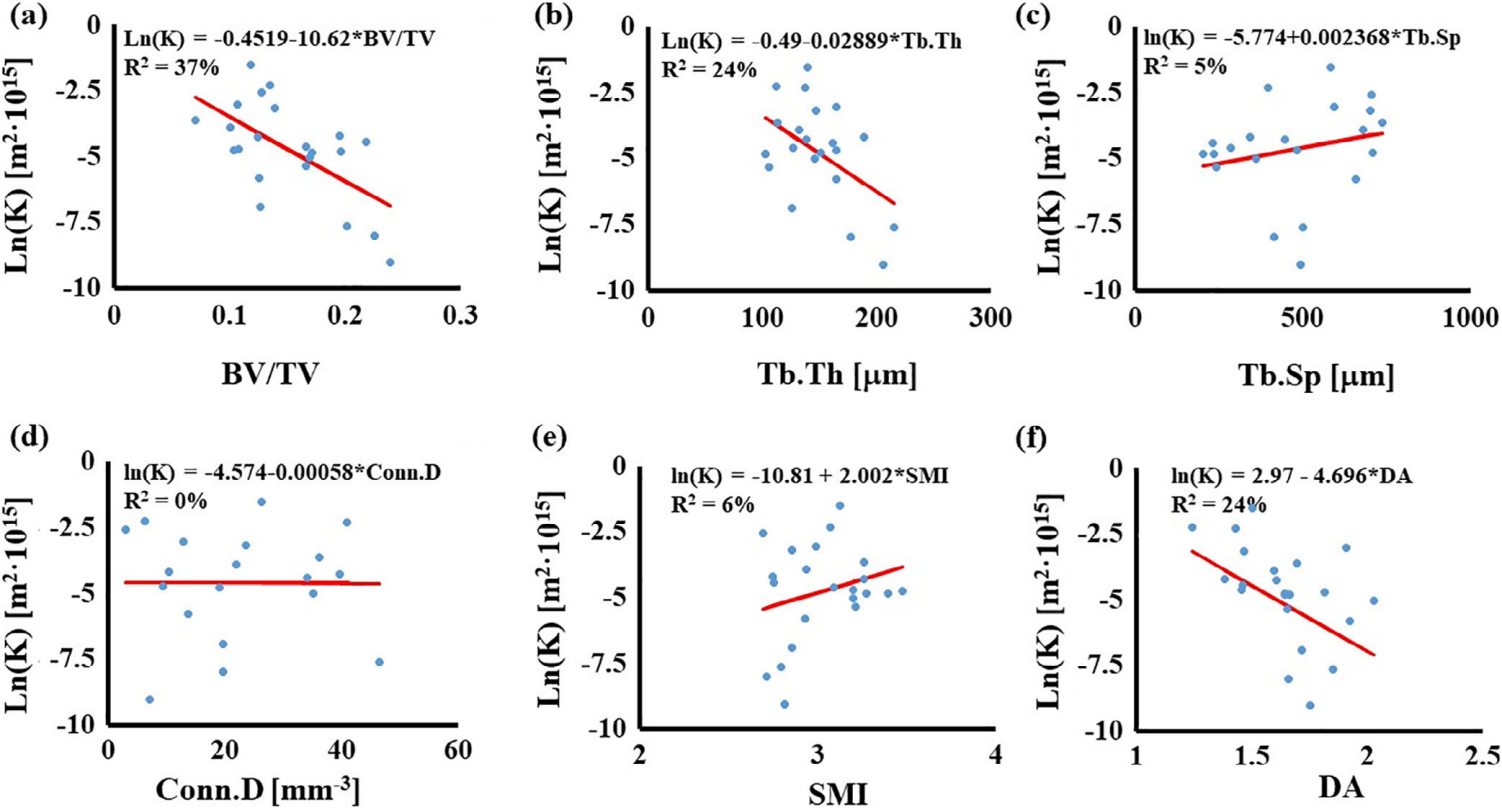
Simple linear regressions for K with morphological parameters: (*A*) relationship with BV/TV; (*B*) relationship with Tb.Th; (*C*) relationship with Tb.Sp; (*D*) relationship with Conn.D; (*E*) relationship with SMI; and (*F*) relationship with DA. In the figures, circles are the experimental points; the continuous line represents the regression model. BV/TV = bone volume over total volume; Conn.D = connectivity density; DA = degree of anisotropy; SMI = structure model index; Tb.Sp = trabecular spacing; Tb.Th = trabecular thickness.

The simulated permeability ranged from 1·10^−19^ to 3.32·10^−16^ m^2^, with a mean value of 2.2·10^−17^ m^2^. Statistically significant correlations were found between K and BV/TV (*R* ^2^ = 37%), Tb.Th (*R* ^2^ = 24%), and DA (*R* ^2^ = 24%) (see Fig. 2A‐F).

**Figure 3:**
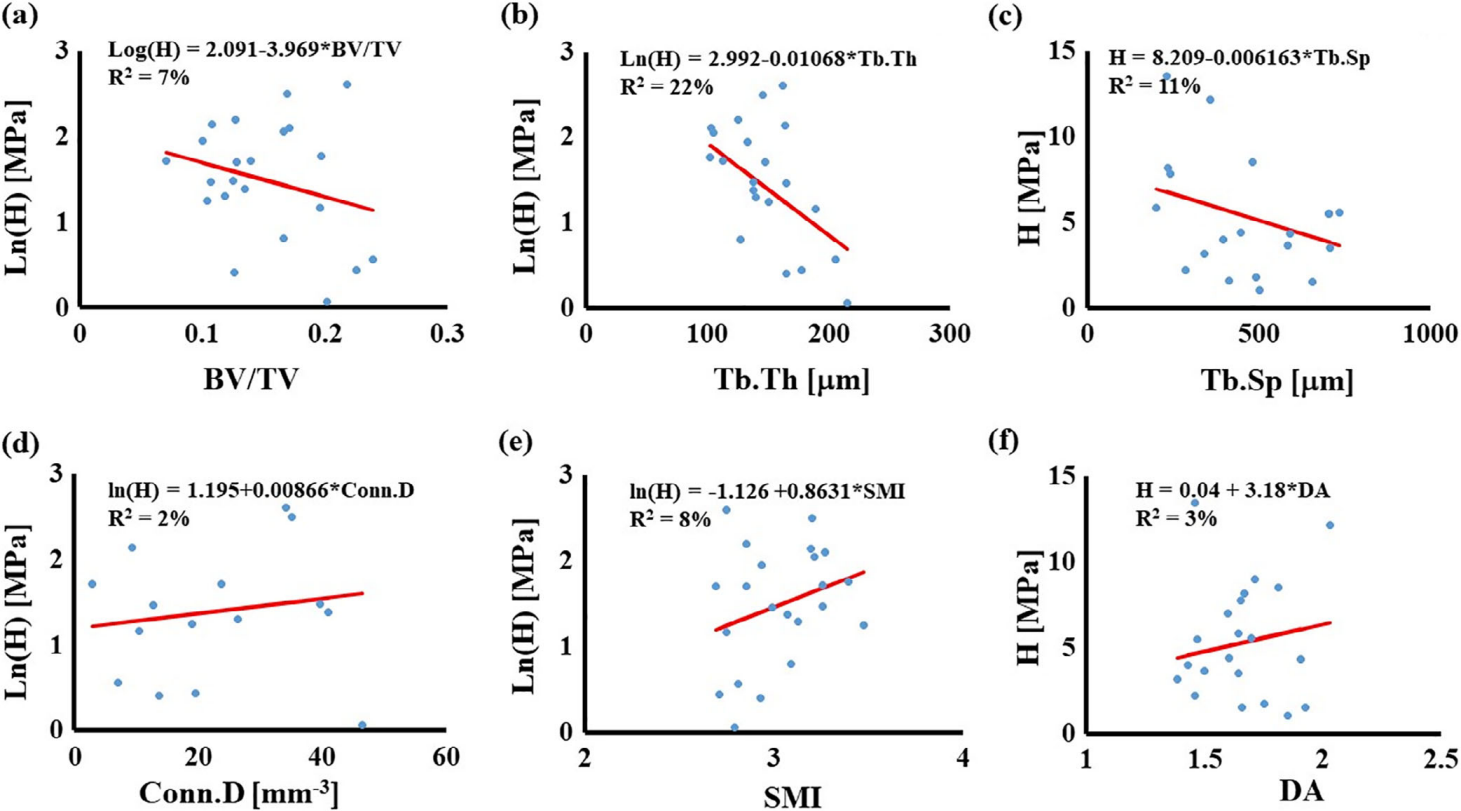
Simple linear regressions for H with morphological parameters: (*A*) relationship with BV/TV; (*B*) relationship with Tb.Th; (*C*) relationship with Tb.Sp; (*D*) relationship with Conn.D; (*E*) relationship with SMI; and (*F*) relationship with DA. In the figures, circles are the experimental points, the continuous line represents the regression model. BV/TV = bone volume over total volume; Conn.D = connectivity density; DA = degree of anisotropy; SMI = structure model index; Tb.Sp = trabecular spacing; Tb.Th = trabecular thickness.

The mean value of the simulated modulus H was 5.27 MPa, and its range spanned from 1.07 to 13.5 MPa. A statistical significant correlation was found between H and Tb.Th (*R* ^2^ = 22%); no other statistically significant relationships were found with other parameters (see Fig. 3A‐F).

The relationships among morphological parameters are summarized in Table 2; BV/TV was positively correlated to Tb.Th (*R* ^2^ = 22%) and negatively correlated to Tb.Sp (*R* ^2^ = 44%); SMI was negatively correlated to Tb.Th (*R* ^2^ = 48%) and positively correlated to Conn.D (*R* ^2^ = 28%). No other statistically significant correlations were found among the rest of the morphological parameters.

**Table 2:** Summary of Regression Analysis Among Morphological Parameters

	Tb.Th	Tb.Sp	Conn.D	SMI	DA
BV/TV	0.22*	0.44*	0.02	0.16	0.02
Tb.Th		0.12	0.09	0.48*	0.08
Tb.Sp			0.15	0.04	0.05
Conn.D				0.28*	0.00
SMI					0.03

Data are reported in terms of coefficient of determination (*R* ^2^).

BV/TV = bone volume over total volume; Conn.D = connectivity density; DA = degree of anisotropy; SMI = structure model index; Tb.Sp = trabecular spacing; Tb.Th = trabecular thickness.

* *p* < 0.05.

Table 3 gives the ordered eigenvectors of six principal components (PC_1_ ~ PC_6_) obtained by PCA along with the sum of six eigenvectors (PC_T_), in which four parameters (Tb.Sp, Conn.D, SMI, and DA) have negative values. Although the first three principal components explain 100% of the total sample variance, all principal components and their aggregate values were considered to manipulate individual parameters in multivariate analysis. For example, the reciprocal of those four parameters were tested, thereby their inverse proportion impacts among others being reflected on the multivariate analysis. As a result, multivariate analysis attempting to relate K and H to six original parameters was conducted, resulting in two linear combinations of ln(K) and ln(H) with *R* ^2^ of 69.85% and 38.60%, respectively, as shown in Table 4. The reciprocal of four parameters (ie, Tb.Sp^−1^, Conn.D^−1^, SMI^−1^, and DA^−1^) yielded better results; both ln(K) and ln(H) were obtained with *R* ^2^ of 74.19% and 46.65%, respectively, as shown in Table 5. We tested various combinations of reciprocal parameters of Tb.Sp, Conn.D, SMI, and DA, and finally found the best set of reciprocal parameters (ie, Conn.D^−1^, SMI^−1^, and DA^−1^), which produced the regression results with the highest *R* ^2^ [ie, 76.48%, 46.86% for ln(K) and ln(H), respectively], as shown in Table 6.

**Table 3:** Summary of Principal component analysis Results for Morphological Variables

Variable	Eigenvectors						
	PC_1_	PC_2_	PC_3_	PC_4_	PC_5_	PC_6_	PC_T_
BV/TV	0.000	0.000	−0.001	0.088	0.025	0.996	1.108
Tb.Th	0.003	0.749	−0.662	0.001	−0.005	−0.001	0.085
Tb.Sp	−0.997	−0.048	−0.059	0.000	0.000	0.000	−1.104
SMI	0.077	−0.661	−0.747	0.003	0.000	−0.001	−1.329
Conn.D	0.000	−0.004	0.001	−0.563	−0.823	0.07	−1.319
DA	0.000	0.002	−0.004	−0.822	0.567	0.058	−0.199
Eigenvalue	89334	2312	420	0.000	0.000	0.000	‐
Proportion	0.970	0.025	0.005	0.000	0.000	0.000	‐
Cumulative	0.970	0.995	1.000	1.000	1.000	1.000	‐

BV/TV = Bone volume over total volume; Conn.D = connectivity density; DA = degree of anisotropy; SMI = structure model index; Tb.Sp = trabecular spacing; Tb.Th = trabecular thickness.

**Table 4:** Results of Multivariate Regression Analysis on K and H by Original Observations

Response	Coefficients of independent parameters							*R* ^2^	*p* value
	BV/TV	Tb.Th	Tb.Sp	Conn.D	SMI	DA	Intercept		
ln(K)	−52.20	0.0276	−0.00402	0.0154	−1.11	−4.38	11.10	69.85%	0.002
ln(H)	−5.29	−0.01323	−0.00123	−0.00212	−0.96	1.11	6.04	38.60%	0.191

BV/TV = bone volume over total volume; Conn.D = connectivity density; DA = degree of anisotropy; SMI = structure model index; Tb.Sp = trabecular spacing; Tb.Th = trabecular thickness.

**Table 5:** Results of Multivariate Regression Analysis on K and H by Reciprocal of Tb.Sp, Conn.D, SMI, and DA

Response	Coefficients of independent parameters								*p* value
	BV/TV	Tb.Th	Tb.Sp^−1^	Conn.D^−1^	SMI^−1^	DA^−1^	Intercept	*R* ^2^	
ln(K)	−57.1	0.027	1563	−2.20	20.00	7.94	−15.32	74.19%	0.001
ln(H)	−9.41	−0.01174	452	1.69	15.99	−3.43	0.25	46.65%	0.082

BV/TV = bone volume over total volume; Conn.D = connectivity density; DA = degree of anisotropy; SMI = structure model index; Tb.Sp = trabecular spacing; Tb.Th = trabecular thickness.

**Table 6:** Results of Multivariate Regression Analysis on K and H by Reciprocal of Conn.D, SMI, and DA

Response	Coefficients of independent parameters							R^2^	*p* value
	BV/TV	Tb.Th	Tb.Sp	Conn.D^−1^	SMI^−1^	DA^−1^	Intercept		
ln(K)	−44.99	−0.0006	−0.00572	9.83	11.1	11.56	−6.22	76.48%	0.000
ln(H)	−5.65	−0.01973	−0.001596	5.10	13.34	−2.44	2.89	46.86%	0.080

BV/TV = bone volume over total volume; Conn.D = connectivity density; DA = degree of anisotropy; SMI = structure model index; Tb.Sp = trabecular spacing; Tb.Th = trabecular thickness.

## Discussion

The rigidity and permeability of cancellous bone provide crucial information on the mechanical behavior of the vertebra that can be exploited to design treatments to pathological conditions such as osteoporosis, age‐related bone loss, and vertebral compression fractures.^1^, ^2^, ^3^, ^5^, ^8^, ^9^ The objective of this study was to assess the power of morphological parameters as predictors of local values vertebral mechanical properties. The values K and H were obtained from indentation tests. Then, statistical analyses were conducted to establish empirical models relating the predictors BV/TV, Tb.Th, Tb.Sp, SMI, Conn.D, and DA to the dependent variables K and H. Supported by a PCA approach, we found that linear combinations of the six morphological predictors correlate up to approximately 76% to the values of K, whereas weaker relationships (*R* ^2^ ~46%) are found for the case of H.

Indentation is a common technique for testing the mechanical behavior of materials at length scales ranging from nanometers to millimeters.^26^, ^31^, ^32^, ^33^ Being able to provide a “local” measurement of the sought properties, indentation is particularly appealing for mapping local properties in heterogeneous media. In addition, by changing the size/shape of the indentation tip or the indentation load, one can test and characterize a range of hierarchical features within the same tissue.^34^ In this study, we used a single indenter of 5‐mm diameter on bone samples whose average trabecular space ranged from 200 to 1500 μm. This proportion of the indenter radius to the trabecular space resembles that successfully used by Oyen and colleagues, who, when testing bones with pore diameters ranging from 10 to 60 μm, used indenters of contact sizes ranging from 230 to 290 μm.^26^

The measured values of the bone morphological parameters were consistent with those reported in similar studies on human cancellous bone.^18^, ^19^ The values of K we measured were smaller than those previously reported in human vertebras.^9^, ^13^, ^14^ This might partly be because, compared with previous studies, the sample size of this contribution was limited and might not have captured the full spectrum of values of mechanical properties of vertebral cancellous bone. In any case, direct comparison with the results of those studies is not possible as they measured permeability as a bulk property of the entire sample: K was determined forcing fluid through core samples or entire vertebras. A direct comparison with the results reported in the microcomputational study by Teo and Teoh is also not possible because the major difference between the average SMI of their samples, being almost 3 times smaller than that measured in this study.^17^ Such a discrepancy indicates that there are substantial differences in the structural organization of the vertebral samples used by Teo and Teoh and those investigated in this contribution. Instead, our findings can be related to those obtained by Oyen and colleagues using micro‐indentation and reporting values of K two orders of magnitude smaller than those measured in the current study.^26^ This is expected in light of the differences in terms of bone density of the bone samples used in the two studies. The specimens used in this study were less dense, with a volumetric fraction of bone accounting for only 15% of the total volume. This resulted in an average intertrabecular spacing of 545 μm, significantly higher than the 200‐μm interpore spacing of the specimens used by Oyen and colleagues. Such morphological differences would also explain the fact that the values of shear modulus measured in the equine specimens were two orders of magnitude larger than the values of aggregate modulus measured in this study.

Results from univariate analysis suggested significant correlations between BV/TV and trabecular thickness and spacing. Additionally, SMI was found to correlate with the trabecular thickness and Conn.D. It was also found that the morphological parameters, if considered individually, are weak predictors of vertebral bone mechanical properties. For the case of K, the only statistically significant correlations were found with BV/TV (*R* ^2^ = 37%), Tb.Th (*R* ^2^ = 24%), and DA (*R* ^2^ = 24%). Although it might be difficult to provide a mechanistic explanation why the permeability is negatively correlated to DA, it seems intuitive to find that K decreases as the bone volumetric density or the thickness of the trabecula increases. In fact, similar—although weaker—correlations have already been suggested.^17^ The modulus H was weakly correlated to the trabecular thickness (*R* ^2^ = 22%). Studies on the length scale of the whole vertebra have shown stronger correlations (*R* ^2^ > 50%) among μCT‐measured morphological parameters and vertebral strength and stiffness.^35^, ^36^ Also, no significant correlation was found between H and BV/TV, which intuitively should dominate the stiffness of the bone. Further studies, increasing the sample size, are needed to verify if such a relationship may exist.

The primary aim of PCA is to reduce the dimensionality of a data set, while retaining as much as possible of the variation present in the data set.^37^ However, in this study, we implemented PCA for the purpose of data interpretation (eg, investigating proportional or inverse proportional relations to others) rather than data reduction. Manipulating parts of the morphological parameters by the results of PCA (ie, reciprocal of Conn.D, SMI, and DA) finally yielded regression models showing about 9.5% and 21% higher correlations with K and H, respectively, than those with original parameter values. In sum, this validates our research hypothesis that a multivariate statistical model with PCA, by including the contribution of morphology parameters at once, can provide strong correlation with the permeability of the vertebral cancellous bone. It should be also noted that such a result is comparable to the highest reported correlation of K with a bone structural parameter in vertebras,^9^ the bone porosity (*R* ^2^ = 66% to 82%), which was measured via a more invasive approach based on the Archimedes' principle.^13^

Limitations in the experimental methods adopted in this study must be noted. In the simulations, the mechanical properties of the vertebral bone were assumed to be isotropic and homogeneous across the entire computational domain. Previous studies have shown that both elastic modulus and hydraulic permeability of the vertebra are transversely isotropic.^9^, ^13^, ^38^ Besides, it is expected that such properties vary within the sample according to the local morphology of the bone. Future studies are needed to finely calculate bone permeability and strength considering a transverse isotropic model. However, to appraise the implications of these simplifications on the estimation of K and H, we investigated the stress field and the fluid flow field within the region of indentation and in its surrounding. Within the region of indentation, we found that the magnitudes of mechanical stress (approximately 10^−1^ MPa) and fluid flow (approximately 10^−3^ mm^3^ s^−1^) in the axial direction (along the direction of the indentation) were over one order of magnitude larger than those found in the transverse direction. Note that a larger fluid flux along the axial direction is especially expected in vertebral bone regions adjacent to vertebral endplates, where the tissue structure for rabbit animal models has been found to be characterized by the presence of vertical canals.^39^ These findings suggest that the reaction force at the indenter, used to determine K and H, is mostly caused by bone deformation and a fluid redistribution occurring in the axial direction, which, in their turn, depend on the axial components of the aggregate modulus and permeability. Hence, the estimated values of K and H should be regarded as the axial components of the permeability and aggregate modulus of the bone, and the error in their estimates (caused by the contribution of the transverse components) should be minimal. Similarly, the magnitudes of the effective stress and fluid flow in the region of indentation (extending from the contact area with the indenter through the entire thickness of the sample) were two orders of magnitude larger than those found in the rest of the computational domain (approximately 10^−3^ MPa and approximately 10^−5^ mm^3^/s^−1^, respectively). This indicates that the bone tissue surrounding the indentation region did not significantly contribute to the reaction force at the indenter. Moreover, the biphasic model used for describing the mechanical behavior of the bone assumed fluids to be inviscid. The viscosity of the bone marrow permeating the cancellous bone can be over two orders of magnitude larger than that of the water.^40^ This might have had an effect on the evaluation of the hydraulic permeability, and thus promotes the future development of a more accurate theoretical formulation for the biphasic mechanical behavior of the vertebral cancellous bone, specifically accounting for the viscosity of its bone marrow. In addition, μCT imaging is limited to ex vivo applications or in vivo studies on relatively small animal models. Future studies are needed to establish whether image analysis of CT scans, which are routinely performed in clinical settings, can deliver bone morphological features providing good correlates with local values of K and H. Finally, the inclusion of gender, spinal level, age, clinical history, and previous pharmacological therapy as independent random variables in the statistical approach we proposed might improve the predictive power of the empirical models. However, the sample size used in this study was limited to 23 specimens. This did not allow us to have a wide enough spectrum for these additional parameters to make them relevant for the present analysis. Future studies, including a larger sample size, may certainly benefit from including these additional descriptors of bone quality for the purpose of statistical inference.

In conclusion, in this study we provided statistical relations between local values of elasticity and permeability, and experimental parameters descriptive of the structure of the cancellous bone of the vertebral body. By adopting an approach supported by PCA, we were able to determine a multivariate statistical model providing strong correlation of K with respect to linear combinations of parameters characterizing bone morphology. Marginal correlation of H with respect to these parameters was also observed. The experimental results of this study, which were generated on an extensive range of bone morphological parameters, can be deployed in computational mechanics frameworks for predicting resistance to fracture and/or capability of withstanding a specific surgical treatment (eg, internal fixation versus bone cement augmentation).

## Disclosure

The authors have nothing to disclose.
